# Elabela Attenuates Doxorubicin-Induced Oxidative DNA Damage and Apoptosis in Rat Left Ventricular Myocardium

**DOI:** 10.3390/biomedicines13102407

**Published:** 2025-09-30

**Authors:** Katarzyna Matusik, Katarzyna Kamińska, Izabela Janiuk, Kaja Kasarełło, Maja Owe-Larsson, Daniel Dylko, Agnieszka Cudnoch-Jędrzejewska

**Affiliations:** 1 Department of Experimental and Clinical Physiology, Laboratory of Centre for Preclinical Research, Medical University of Warsaw, Banacha 1b, 02-097 Warsaw, Poland; katarzyna.matusik@wum.edu.pl (K.M.); maja.owe-larsson@wum.edu.pl (M.O.-L.);; 2 Department of Histology and Embryology, Center of Biostructure Research, Medical University of Warsaw, Chałubińskiego 5, 02-004 Warsaw, Poland; izabela.janiuk@wum.edu.pl

**Keywords:** apelinergic system, cardiotoxicity, doxorubicin, elabela, oxidative stress

## Abstract

**Background:** Doxorubicin, a widely used chemotherapeutic agent, has been shown to increase reactive oxygen species (ROS) levels, disrupting cellular homeostasis not only in cancer cells but also in healthy tissues, particularly in cardiomyocytes, which leads to chemotherapy-induced cardiotoxicity. Therefore, new strategies are continually being explored to mitigate these adverse effects. One such approach is the use of additional substances with cardioprotective properties during doxorubicin therapy. A promising candidate is elabela, a peptide of the apelinergic system, which may exert protective effects against doxorubicin-induced oxidative stress in cardiomyocytes. **Objectives**: This study aims to evaluate the modulatory effects of elabela on oxidative stress markers, malondialdehyde (MDA) and 8-hydroxy-2′-deoxyguanosine (8-OHdG) in the left ventricle of the myocardium following chronic doxorubicin administration in rats. **Material and Methods:** 32 male, 12-week-old Sprague-Dawley rats (SPRD) were randomly assigned to four experimental groups. For 28 days, all animals received continuous infusions (2.5 μL/h) via subcutaneously implanted osmotic pumps of 0.9% NaCl or elabela (40 μg/kg body weight/day or 200 μg/kg body weight/day). Simultaneously, animals were injected intraperitoneally 4 times at weekly intervals with 0.9% NaCl or DOX (3.5 mg/kg body weight). Next, the animals were sacrificed, and left ventricular (LV) cardiac tissue was collected for further analysis. MDA and 8-OHdG and elabela level in LV lysate were assessed by ELISA. The *Ela* expression in LV was quantified by Real-Time PCR. The TUNEL assay, labeled with a 5′-triphosphate strand, was used to assess the degree of apoptosis. **Results:** DOX treatment decreased both the *Ela* expression and elabela levels in the LV. Elabela administration at a dose of 200 µg/kg body weight/day significantly decreased ELA levels and *Ela* expression compared to the control group. The level of 8-OhdG was unexpectedly decreased in the DOX group compared to controls, while elabela treatment at both doses restored 8-OHdG levels observed in the control group. However, TUNEL staining demonstrated that elabela administration at 200 µg/kg body weight/day reduced the number of apoptotic cardiomyocytes compared to the DOX-only group, indicating a protective effect against DOX-induced apoptosis. The lower dose of 40 µg/kg body weight/day showed a moderate, non-significant attenuation of apoptosis. **Conclusions:** Elabela showed a protective effect against DOX-induced cardiomyocyte apoptosis in the LV by promoting processes that reduce oxidative stress in cardiac cells.

## 1. Introduction

Oxidative stress (OS) is a pathological state characterized by an imbalance between increased levels of reactive oxygen species (ROS) and insufficient antioxidant defense mechanisms [1]. The consequences of this process include lipid peroxidation and DNA damage, reflected, among others, in elevated levels of malondialdehyde (MDA) and 8-hydroxy-2′-deoxyguanosine (8-OHdG), compounds widely recognized as biomarkers of OS [2]. Both are widely used due to their specificity and reliability in reflecting oxidative injury [3,4]. Elevated levels of these markers have been associated with various diseases, including cancer, cardiovascular and neurodegenerative disorders, and skin conditions such as vitiligo [5,6]. Ultimately, excessive ROS activity contributes to irreversible cellular damage and cell death [7,8].

The area of medicine where the knowledge of OS metabolism and ways to defend against it is most critical is cancer therapy. This is due to the activity of many of the chemotherapeutic agents, which results in the production of high amounts of ROS as part of their anticancer effects. Anthracyclines (ANTs) are commonly used in chemotherapy because of their strong effectiveness, making them widely recognized as a standard treatment for several types of cancer, including breast, lung, and ovarian cancer [9]. There are two main mechanisms of ANT action: generation of ROS and intercalation of DNA and RNA [10]. However, these same mechanisms are also responsible for the most significant drawback of ANTs, as a result of its unselective action, not only on cancer cells but also healthy cells. Healthy heart cells are susceptible to ROS-induced damage due to their low antioxidant defense, while the high ATP demand of cardiomyocytes further increases mitochondrial vulnerability to oxidative injury [11]. In such conditions, this vulnerability leads to oxidative damage of cellular membranes and DNA, resulting in increased lipid peroxidation and genotoxic stress. Finally, this cascade contributes to mitochondrial dysfunction, progressive cardiomyopathy, heart failure, and cardiovascular complications, including anthracycline-induced cardiotoxicity [8].

Doxorubicin (DOX) is one of the most widely used anthracyclines. DOX chemotherapy has been shown to trigger an important pathologically relevant multifactorial pathway leading to the development of cardiomyopathy [12]. This includes widely studied cardiotoxic complications in both animal and human models [8,9,10,11,12,13,14,15,16], but also cardiometabolic problems such as impaired glucose metabolism, insulin resistance and dyslipidemia [15]. Its toxicity is mainly linked to excessive ROS production, driven by redox cycling of its quinone group, mitochondrial electron transport chain disruption, and iron-mediated Fenton reactions [16,17,18,19,20]. ROS-induced OS disrupts cellular homeostasis by affecting autophagy, apoptosis, and cardiac function [2], and contributes to endothelial dysfunction, vascular damage, and ischemia [21]. Koleini and coworkers (2017) [13] concluded that DOX causes dysregulation in autophagy and mitophagy, boosting autophagosome formation through various chemical pathways and autophagy-related genes [13]. Furthermore, apoptosis driven by ROS formation has been identified as a central mechanism in the pathogenesis of doxorubicin-induced cardiotoxicity (DIC) [14]. From earlier studies using the rat model of DIC by Alwaili et al. (2025) [16], high ROS levels were shown to impair myocardial function and were associated with elevated serum levels of inflammatory tumor necrosis factor-alpha (TNF-α) and interleukin-6 (IL-6) [16].

These cytokines, in turn, amplify OS by promoting further ROS generation and mitochondrial dysfunction. A clinical study by Mesquida et al. (2009) [17] on metabolic syndrome found a significant positive correlation between elevated concentrations of the pro-inflammatory cytokines TNF-α and IL-6 and MDA levels [17]. In contrast, elevated levels of 8-OHdG have been associated with inflammation and endothelial dysfunction in the course of cardiovascular disease [18].

There are several cardioprotective strategies to reduce DIC, however, this diversity often reflects the absence of a universally effective approach. Dexrazoxane, an EDTA analog, is the only approved cardioprotector against DIC at cumulative doses ≥300 mg/m^2^, acting as an iron chelator. However, due to its topoisomerase inhibition similar to DOX, it may cause myelosuppression, secondary malignancies, and reduce chemotherapy efficacy. Safety concerns, especially in patients with low LV ejection fraction (LVEF), heart failure (HF), or lower ANT doses, limit its clinical use. Natural compounds like resveratrol, a polyphenol with antioxidant properties, show promise in preclinical models by inhibiting ferroptosis and reducing iron accumulation [19]. Coenzyme Q10, another antioxidant concentrated in mitochondria, may reduce OS markers like 8-OHdG and lipid peroxidation. Although meta-analyses show potential cardioprotection by Q10, clinical data remain limited [20]. Resveratrol and Q10 are promising but require further validation in clinical trials. Since these options are not fully sufficient to reduce OS and cardiac damage, alternative protective strategies are still being explored.

The apelinergic system (AS) consists of apelin and elabela, found in the cardiovascular system, and has attracted increasing interest in the context of DIC. Recent studies have emphasized the potential effectiveness of strategies targeting AS [18]. Preclinical studies indicate that activation of the AS can affect OS: AS activation reduced serum MDA and 8-OHdG level [22], increased heart rate and nitric oxide levels in myocardial damage model [23] and finally modulated pro-inflammatory signaling by inhibiting the inflammatory response thought down-regulating the nuclear factor (NF-κB) pathway and up-regulating the ERK1/2 pathway [24]. Downregulating the AS in animal models has been shown to induce progressive HF and predispose to cardiac OS [25].

Elabela is an endogenous ligand of the APJ receptor, which belongs to the GPCR receptor family. The primary structure of elabela comprises 32 amino acids; however, shorter forms with equivalent activity, such as elabela-22 or elabela-11, have also been identified. Elabela exhibits essential activity during embryonic development, specifically in the formation of the heart and blood vessels [26], and is expressed in only a small number of tissues and organs, such as the kidney and heart [27]. The restricted distribution of elabela may suggest a more specialized function for this ligand, particularly in the cardiovascular and renal systems. The limited expression of elabela may be closely regulated in response to local stress conditions. Furthermore, despite having a common APJ receptor, elabela exhibit different biological properties in terms of OS and doxorubicin-induced cardiomyocyte damage.

Wang et al. (2022) [24] demonstrated in a murine model of DIC that elabela-11 increased mitochondrial membrane potential compared to DOX treatment alone. Moreover, elabela-11 reduced the expression of cleaved caspase-3 and Poly (ADP-ribose) Polymerase (PARP), sensors of DNA strand breaks, and decreased DOX-induced free radical production. Additionally, elabela-11 inhibited MDA formation and enhanced antioxidant enzyme activity [24]. These findings suggest that elabela-11 mitigates DOX-induced cardiac damage by attenuating OS-mediated apoptosis. In the same study, it has been proven that elabela-11 inhibits apoptosis induced by OS in cardiomyocytes damaged by cobalt chloride (CoCl_2_). Also, TUNEL staining clearly indicated that the number of apoptotic cells increased in the DOX-induced group but decreased after concurrent treatment with elabela-11 [28].

Additionally, a positive effect of elabela was observed in restoring autophagic flux in cardiomyocytes by enhancing the activity of transcription factor EB (TFEB), which improves the removal of accumulated and damaged cellular components [27]. Moreover, activation of ERK/MAPK and PI3K/AKT signaling pathways by elabela-11 has resulted in reduction in myocardial fibrosis and vacuolization of cardiomyocytes, leading to suppression of apoptosis [28].

Taken together, these findings suggest that targeting elabela-mediated pathways could reduce oxidative damage, normalize autophagy, and inhibit apoptosis. This might improve the survival of cardiomyocytes and reduce the progression of chemotherapy-induced cardiotoxicity. Considering the above, the present study is designed to investigate the potential cardioprotective effects of elabela by assessing its ability to modulate the level of OS markers, specifically MDA and 8-OHdG, during chronic exposure to DOX. The primary aim is to evaluate whether elabela can effectively mitigate OS as reflected by changes in these biomarkers.

## 2. Materials and Methods

### 2.1. Animals

The studies were conducted using 32 male Sprague-Dawley rats SPRD (weight 250–300 g), 12-week-old. All experimental procedures were approved by the II Local Animal Research Ethics Committee (Application No. WAW2/087/2021, release date: 2 June 2021). The rats subjected to the experiment were bred and maintained at the Central Laboratory of Experimental Animals of the Medical University of Warsaw, Poland in followed conditions: temperature (22–24 °C), light-dark cycle (12:12), and humidity (45–65%) with standard feed pellet (Labofeed B) and water constituted an ad libitum diet. The animals were housed 4 per conventional transparent polycarbonate cages with metal lids (type 1354G). Environmental enrichment was provided in the form of a red cuboid tunnel.

### 2.2. Experimental Design

Animals were randomly assigned to 4 groups (n = 8). On day 1, animals were placed under general anesthesia by intraperitoneal (i.p.) administration of ketamine (75 mg/kg body weight) with xylazine (7 mg/kg body weight and underwent a surgical procedure to implant an osmotic pump (Alzet Corp., Cupertino, CA, USA, model 2ML4) subcutaneously in the intercostal space. After surgery, the animals were kept separately in conventional cages with metal lids for 24 h to prevent mutual removal of sutures and wound damage by other individuals. After separation, individuals were grouped as before. For the following 28 days, rats were receiving continuous infusions (2.5 μL/h) of 0.9% NaCl or elabela (40 or 200 μg/kg b.w. in 0.9% NaCl). Simultaneously, every 7 days, animals were subjected to a total of four i.p. injections (day 1, 8, 15, 22) of 0.9% NaCl (1 mL) or DOX (3.5 mg/kg b.w. in 1ml of 0.9% NaCl, cumulative dose 14 mg/kg body weight month). Table 1 presents the scheme of the experiment.

At the end of the experiment (7 days after the last DOX/NaCl i.p. administration), the animals were anesthetized (i.p. administration of ketamine (75 mg/kg body weight) with xylazine (7 mg/kg body weight)). Next, the hearts were weighted and collected from animals subjected to deep anesthesia (see the weight of hearts in Table S1 in Supplementary Materials.

Next, the hearts were collected from animals subjected to deep anesthesia: chest was opened by longitudinal incision along the left parasternal line. Isolated hearts were rinsed with 0.9% NaCl solution and then placed on a Petri dish. A transverse incision was made halfway between the base and apex of the heart. The basal fragment was divided into left and right ventricles. Afterwards, apexes and remaining LVs were stored separately at −80 °C until further analysis.

### 2.3. Drugs and Reagents

Ketamine hydrochloride and xylazine have been acquired from Centrovet (Białystok, Poland), while elabela (Elabela (19–32), TFA salt, cat. No.: HY-P2106A) and doxorubicin hydrochloride (cat. No.: HY-15142) were acquired from MedChemExpress (Sollentuna, Sweden).

### 2.4. Analysis of Ela Expression in LV

To assess *Ela* expression, apexes of LVs (100 mg) were homogenized in TRIzol^®^ reagent (Ambion, Life Technologies, Foster City, CA, USA), and total mRNA was extracted using the Monarch Total RNA Miniprep Kit (cat. no. T2110S, New England BioLabs, Ipswich, MA, USA) according to the manufacturer’s protocol. RNA concentration was determined using a Smart Spec™ Plus spectrophotometer (Bio-Rad, Hercules, CA, USA). Real-time multiplex transcription-polymerase chain reaction (Real-time PCR) was performed according to Applied Biosystems protocols using the TaqMan^®^ RNA-to-Ct™ 1-Step Kit (cat. no. 4392938, Thermo Fisher). Primers for target genes from Applied Biosystems, Forest City, CA, USA (rat elabela, gene symbol ELA, Sequences (5′ > 3′) Fwd: ACTTCATTCTCGAGTGCCCTTC, Rev: TGGATCCGAAAAGCCATCCAA, Integrated DNA Technologies, Inc.—labeled with FAM reporter dye, primer for the housekeeping gene (Peptidylprolyl isomerase A PpIA: Applied Biosystems; gene symbol PpIA, accession number Rn00690933_m1) labeled with VIC dye, and RNase-free water RNA arrays (Eppendorf, Hamburg, Germany) were used. The total volume of the reaction mixture (10 μL) was dispensed into 96-well barcoded Micro Amp Optical reaction plates (Applied Biosystems) and placed in a ViiA™ 7 Real-Time PCR System thermocycler (Applied Biosystems). Technical replicates (n = 3) were included for each sample. The amplification reaction was performed in 40 cycles at 95 °C for 15 s and 60 °C for 1 min. Relative gene expression was determined by estimating the delta delta cycle threshold (ΔΔCt) by relative quantification to the PpIA endogenous control.

### 2.5. MDA, 8-OHdG and ELA Levels (Enzyme-Linked Immunoassays Analysis, ELISA)

100 mg of remaining LVs fragments were homogenized with 900 μL of PBS (phosphate-buffered saline) and protease inhibitors (Protease Inhibitor Cocktail, Sigma-Aldrich, St. Louis, MO, USA), using a Tissue Lyser LT (Qiagen, Germantown, MD, USA). Next, homogenates were centrifuged, and subsequently the supernatants were collected and subjected to analysis using commercial ELISA kits, in accordance with the instructions provided by the manufacturer: (1) MDA Double Antibody Sandwich Rat Malondialdehyde (MDA) ELISA Kit, ca.no. EIA06027r; Wuhan Enlibio Biotech Co., Ltd., Wuhan, China, (2) 8-OHdG: Double Antibody Sandwich Rat 8-hydroxy-2-deoxyguanosine (8-OHdG) ELISA Kit, ca.no. EIA05009r; Wuhan Newqidi Biotech Co., Ltd., Wuhan, China (duplicate measurements for each well, the manual is available directly from manufacturer), (3) Elabela: Double Antibody Sandwich Rat Elabela (ELA) ELISA Kit, ca.no. EIA 09766r; Wuhan Enlibio Biotech Co., Ltd., Wuhan, China (duplicate measurements for each well) Representative calibration plots (standard curves) for each analyte are provided in the Supplementary Materials (Figure S1: MDA, Figure S2: 8-OHdG, Figure S3: ELA).

### 2.6. Apoptosis Analysis (Terminal Deoxynucleotidyl Transferase dUTP Nick End Labeling, TUNEL)

Three samples of frozen sections of 10 μm each were prepared from the LV from each individual, cut every 150 µm, and analyzed in five randomly selected fields. The TUNEL assay was performed using the Elabscience^®^ One-step TUNEL In Situ Apoptosis Kit (HRP-DAB Method), according to the manufacturer’s instructions (Elabscience, Houston, TX, USA). The number and percentage of TUNEL-positive cardiomyocytes were counted with the use of a microscope (Nikon) with an eyepiece grid (magnification ×400) and the Opta-Tech 6.0 software (Opta-Tech, Warsaw, Poland). Five fields per sample were analyzed. The images were examined with the use of the QuPath software version 0.5.1 (Belfast, Northern Ireland, UK).

### 2.7. Statistical Analysis

The distribution of continuous variables was analyzed using the Shapiro–Wilk test. For normally distributed variables, the mean and the standard deviation were calculated; if not, the median and the 25th and 75th percentiles (Q1 and Q4) were calculated. ANOVA test was used for comparison of the normally distributed continuous variables; if not, the Kruskal–Wallis test with Holm–Sidak’s or Dunn post hoc test was used. The significance level was set at 0.05. Analyses were performed using the GraphPad Prism software (version 10.4.1, Inc., San Diego, CA, USA). Significance levels were marked on figures as follows: * *p* < 0.05; ** *p* < 0.01, *** *p* < 0.001, **** *p* < 0.0001.

## 3. Results

### 3.1. Elabela Levels in LV

**Figure 1:**
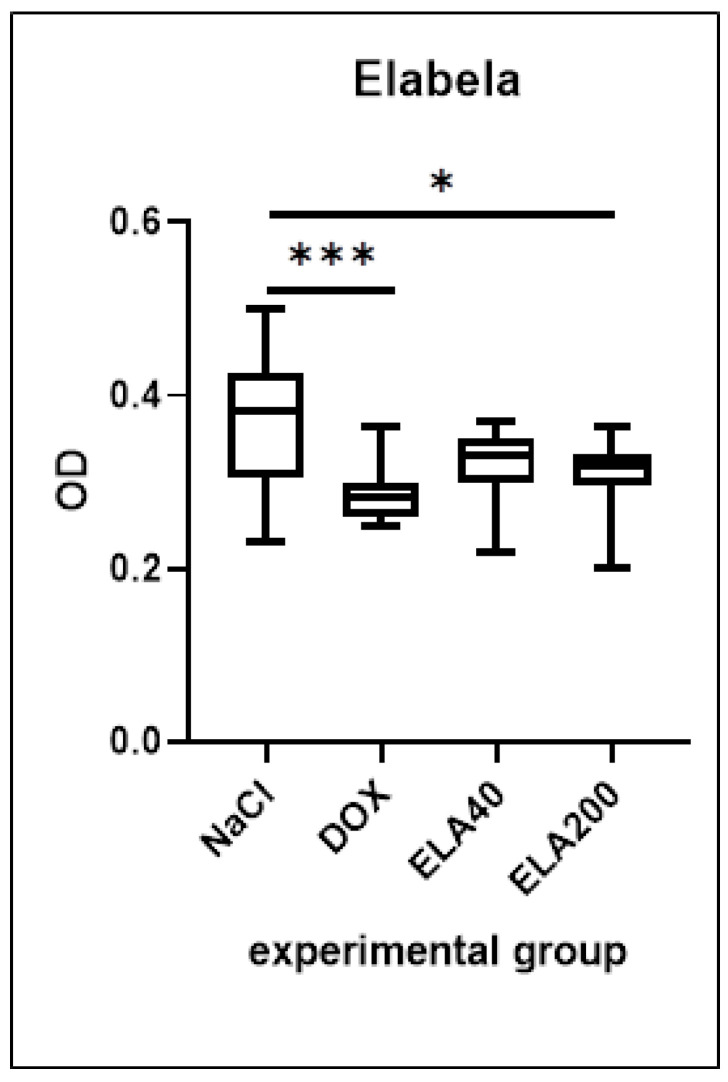
Levels of elabela in the LV of rats, presented as OD (optical density). Values are presented as medians with interquartile ranges (IQR). Statistical analysis: one-way ANOVA with Sidak’s post hoc test. * *p* < 0.05; *** *p* < 0.001.

The DOX group showed a significant reduction in elabela levels in the LV compared to the control group (*** *p* = 0.0003). Elabela administration showed tendencies to increase the Ela levels, compared to DOX groups, but the results are not significant. Moreover, elabela levels after administration of ELA at a higher dose (ELA200) were significantly lower than in the control group (Figure 1).

### 3.2. Ela Expression in LV

**Figure 2:**
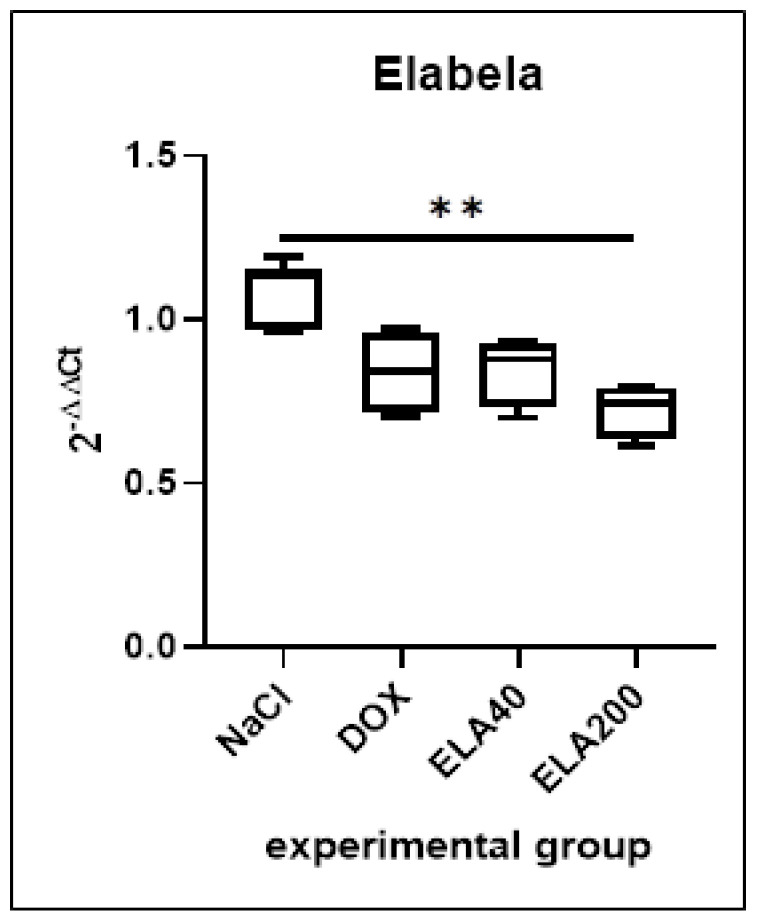
Relative expression of *Ela* (2^−ΔΔCt^) in the LV of rats. Values are presented as medians with interquartile ranges (IQR). Statistical analysis: Kruskal–Wallis test with Dunn’s post hoc test. ** *p* < 0.01.

The DOX group showed a tendency to decrease the *Ela* expression compared to the control group. No significant increase in expression was observed after elabela administration in both doses (ELA40 or ELA200) compared to the DOX group. Additionally, *Ela* expression was significantly lower after administration of the higher elabela dose (ELA200) compared to the control group (** *p* = 0.0086) (Figure 2).

### 3.3. 8-OHdG Level in LV

**Figure 3:**
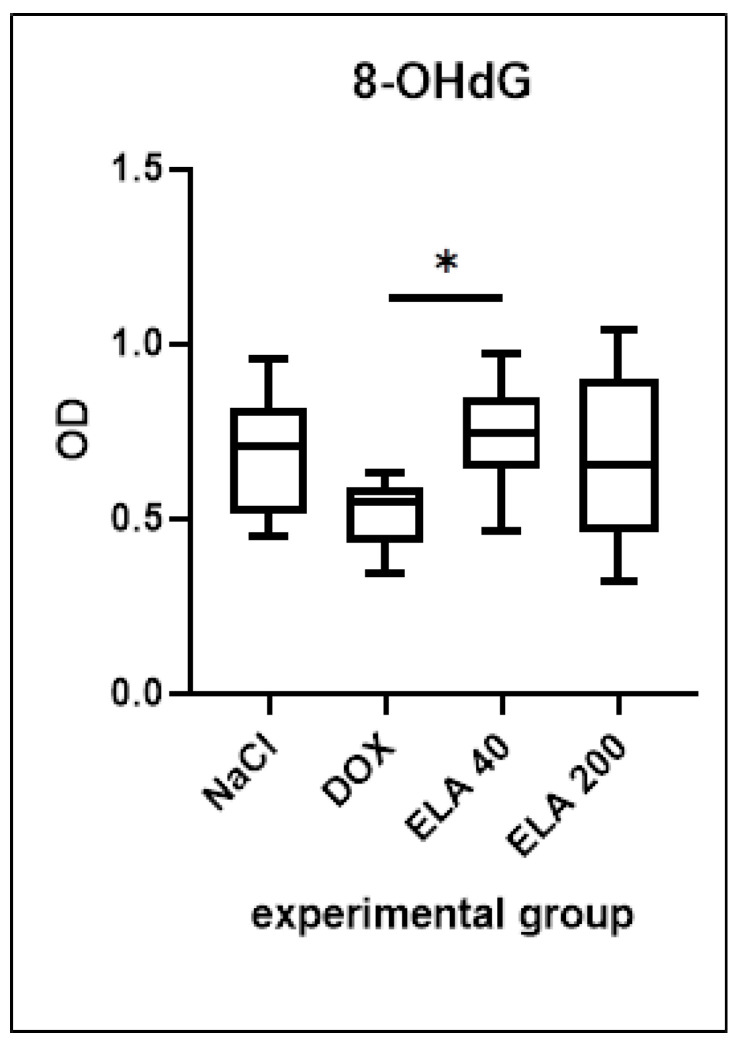
Levels of 8-OHdG in the LV of rats, presented as OD (optical density). Values are presented as medians with interquartile ranges (IQR). Statistical analysis: one-way ANOVA test with Holm–Sidak’s post hoc test. * *p* < 0.05.

The DOX group showed a tendency to reduce the 8-OHdG levels in the LV compared to the control group. There was a significant increase in 8-OHdG levels in the LV of elabela-treated rats (lower dose, ELA40) compared to the DOX group (* *p* = 0.0482), and a visible tendency of such increase for the higher dose of elabela (ELA200) compared to DOX group. No other significant differences were observed between the groups (Figure 3).

### 3.4. MDA Level in LV

**Figure 4:**
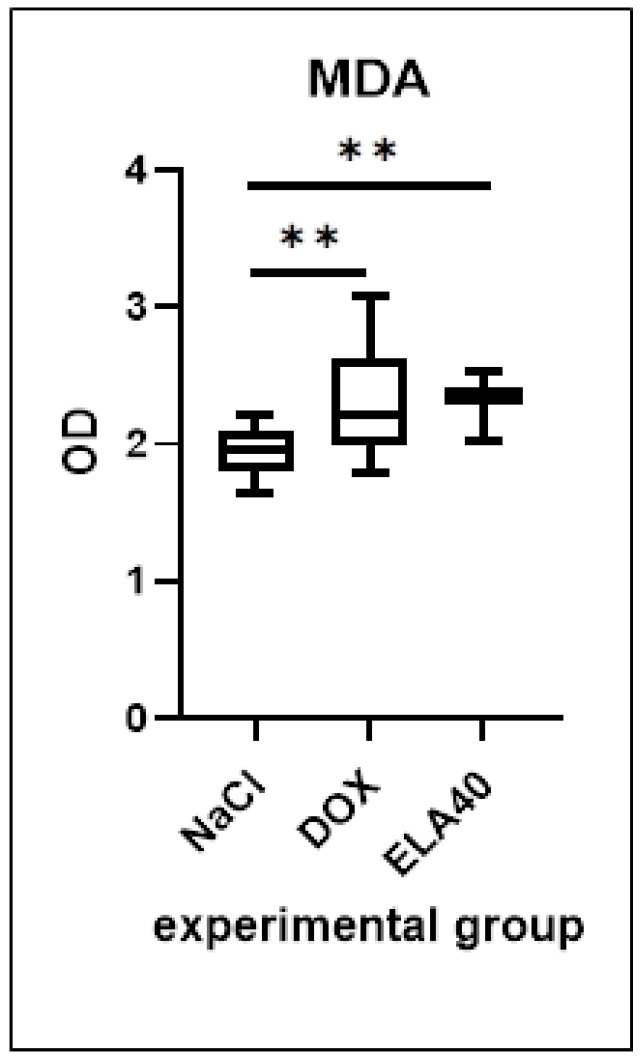
Levels of MDA in the LV of rats, presented as OD (optical density). Values are presented as medians with interquartile ranges (IQR). Statistical analysis: one-way ANOVA test with Sidak’s post hoc test. ** *p* < 0.01.

Both the DOX group and elabela (ELA40) groups showed a significant increase in MDA levels in the LV compared to the control group (** *p* = 0.0032). No significant differences were observed between the elabela-treated group (ELA40) and the DOX group (Figure 4).

### 3.5. Apoptosis Analysis Using the TUNEL Assay

**Figure 5:**
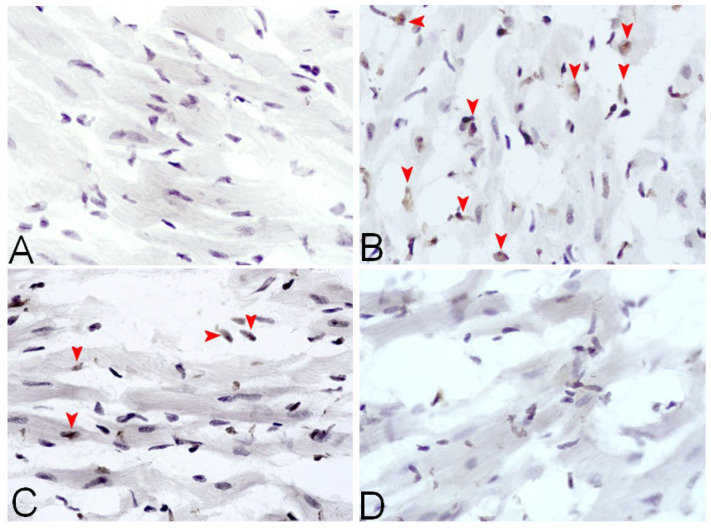
TUNEL assay for apoptotic cardiomyocytes in the LV of (**A**) rats treated with 0.9% NaCl only (control group), (**B**) rats treated with DOX, (**C**) rats treated with doxorubicin and elabela (40 μg/kg b.w.), (**D**) rats treated with DOX and elabela (200 μg/kg/b.w.). Figure shows representative images for each group. The red arrows indicate apoptotic cells. Magnification ×400.

**Figure 6:**
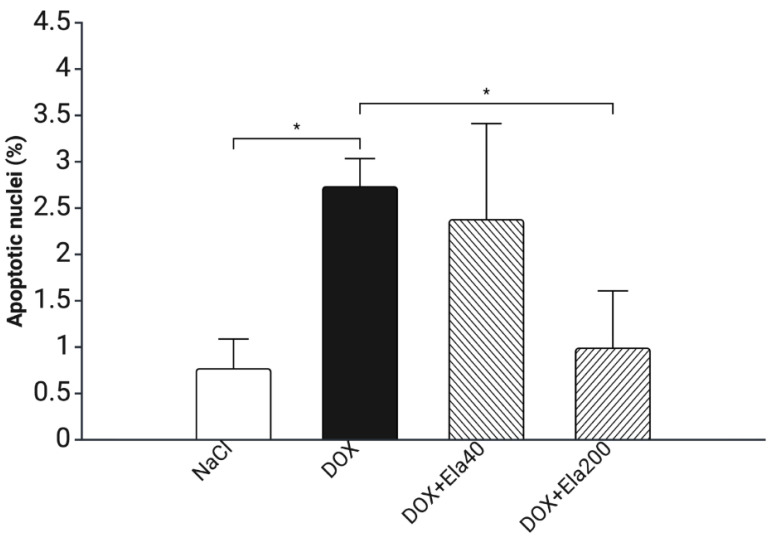
The effect of Elabela on the apoptosis of myocardial cells caused by chronic doxorubicin administration in rats. NaCl—rats treated with saline only (control group), DOX—rats treated with doxorubicin, DOX + ELA40—rats treated with DOX and Elabela (40 µg/kg b.w.), DOX + Ela200—rats treated with DOX and Elabela (200 µg/kg b.w). * *p* < 0.05 (*t*-test). Created in BioRender. Owe-larsson, M. (2025) https://BioRender.com/wc21xhc (accessed on 1 September 2025).

In the control group, there were no visible apoptotic cells. The DOX group presented the most numerous apoptotic cells in the sections. After elabela administration, there was a visible reduction in the number of apoptotic cells in the LV sections, but there was a greater reduction after the administration of the higher dose of elabela (ELA200) (Figure 5, Table 2, Figure 6).

## 4. Discussion

In our study, chronic administration of DOX showed the tendency to reduce *Ela* expression in the LV compared to the control group. This finding is consistent with existing literature demonstrating DOX’s ability to induce OS and apoptosis in cardiomyocytes, which can downregulate genes associated with cardiac protection, including those encoding peptides such as elabela [29]. Interestingly, co-administration of elabela at a dose of 40 µg/kg body weight day ×28 did not reverse this effect, while the higher dose (200 µg/kg body weight day ×28) appeared to further decrease *Ela* expression. This paradox may be explained by a feedback or compensatory suppression mechanism, where exogenous peptide supplementation leads to downregulation of endogenous gene transcription, a phenomenon previously described in the AS under conditions of prolonged ligand exposure [30,31,32,33].

Chronic administration of DOX significantly reduced elabela levels in the LV tissue compared to the control group. Co-administration with elabela did not fully reverse this effect; neither the 40 µg/kg body weight day (ELA40) nor the 200 µg/kg body weight day (ELA200) dose led to a statistically significant increase in peptide levels compared to the DOX group. These findings suggest that prolonged DOX exposure reduces endogenous elabela content in cardiac tissue, and exogenous supplementation, even at higher doses, is insufficient to fully counteract this decline. It is possible that limited peptide retention or enhanced degradation under OS conditions may reduce the effectiveness of substitution therapy, particularly in the context of DOX-induced cardiotoxicity.

Despite a clear increase in apoptosis observed in the group receiving DOX alone, the level of 8-OHdG, a marker of oxidative DNA damage, was non-significantly reduced in this group compared to the control group. Lower dose of elabela (ELA40) administration significantly increased the 8-OhdG levels compared to the DOX group, and a similar tendency was observed after higher elabela dose (ELA200) administration. At the same time, a marked increase in MDA levels was recorded in the DOX-only group and elabela-treated rats, compared to controls, indicating intensified lipid peroxidation processes.

The obtained results may indicate several potential biological mechanisms. One possible explanation is the selective elimination of cells exhibiting the highest oxidative DNA damage. DOX, through the generation of ROS, activates pathways leading to apoptosis, including the activation of the p53 pathway and the caspase cascade, resulting in the death of cells with severe genetic material damage [32,34,35]. As a result, cells with the highest levels of DNA damage may be rapidly eliminated and thus are not represented in the biological material used for the analysis of 8-OHdG levels. Therefore, the measurement of this marker may primarily reflect the state of surviving cells characterized by relatively less DNA damage. Such a mechanism of selective elimination of cells with DNA damage has been described in oxidative damage models and is well documented in the context of chemotherapeutic toxicity [36].

Alternatively, the observed lack of increase in 8-OHdG levels alongside elevated MDA levels may suggest that ROS generated by DOX primarily damage lipid structures, especially within cell and mitochondrial membranes, and to a lesser extent, directly damage DNA. Lipid peroxidation damage is a key element of DOX cardiotoxicity mechanisms and can lead to mitochondrial dysfunction and cell death [37]. Similar discrepancies between markers of DNA and lipid damage have been previously described in studies on oxidative toxicity in heart and liver tissues [38].

Additionally, the timing of sample collection cannot be excluded as a factor influencing the results. The level of 8-OHdG may undergo dynamic changes due to DNA repair mechanisms such as base excision repair and removal of oxidized nucleotides from cells [39]. Repair and elimination of 8-OHdG from DNA may lead to its decreased concentration in the analyzed material, especially in chronic models where damage and regenerative processes occur simultaneously [32].

These results indicate that different forms of oxidative damage to DNA and lipids may occur with varying intensities depending on the mechanism of action of the cytotoxic agent, such as chronic exposure to DOX. Simultaneous analysis of 8-OHdG and MDA levels allows for better differentiation of the oxidative response character in the tissue and provides a more comprehensive picture of the processes of damage and cellular adaptation.

The anti-apoptotic effect was further demonstrated by the TUNEL assay: while DOX induced significant apoptosis in cardiomyocytes, co-administration of elabela at 40 µg/kg body weight day ×28 moderately attenuated this response, and the higher dose completely suppressed it. Elabela is known to modulate apoptotic pathways via activation of PI3K/Akt and ERK1/2 signaling, inhibition of mitochondrial-mediated cell death, and reduction in intracellular ROS. These mechanisms likely converge to protect cardiomyocytes from DOX-induced damage.

Taken together, reduction in oxidative stress and apoptosis may provide evidence that elabela protects against DOX-induced cardiotoxicity through dual mechanisms: inhibition of ROS-mediated damage and suppression of cell death.

Our findings are consistent with previous reports on the antioxidative and cardioprotective properties of elabela. For instance, elabela has been shown to reduce ROS generation, MDA levels, and apoptosis in cardiomyocytes subjected to ischemia–reperfusion injury via PI3K/Akt activation [31]. In DOX-induced cardiac injury models, the elabela-11 fragment preserved myocardial integrity by modulating the PI3K/Akt and ERK/MAPK pathways, thereby attenuating OS-induced apoptosis [30]. Moreover, elabela was found to alleviate DOX-induced OS and ferroptosis in aortic fibroblasts through upregulation of the KLF15/Nrf2/SLC7A11/GPX4 axis, reducing ROS and lipid peroxidation [30]. In diabetic cardiomyopathy models, elabela exerted similar protective effects via SIRT3/Foxo3a pathway activation, leading to reductions in MDA, 4-HNE, and myocardial apoptosis [33].

Elabela’s cardioprotective effect in our model may be explained by several complementary mechanisms. Activation of the APJ receptor can stimulate the PI3K/Akt pathway, leading to inhibition of pro-apoptotic signaling, stabilization of mitochondrial integrity, and reduced caspase activation. In parallel, elabela may enhance eNOS-derived nitric oxide (NO) production, thereby activating the sGC–cGMP–PKG cascade, improving microvascular function, and promoting antioxidant defense through Nrf2-dependent induction of enzymes such as SOD2 and HO-1. Moreover, APJ receptor signaling could attenuate the activity of NADPH oxidases (NOX2/NOX4), limiting primary ROS generation and subsequent oxidative injury. Together, these mechanisms provide a coherent explanation for the observed reduction in lipid peroxidation (MDA), oxidative DNA damage (8-OHdG), and apoptosis in the myocardium of rats treated with elabela during chronic DOX administration. Although direct evidence for these pathways could not be obtained due to limited material, our findings are consistent with previously reported cardioprotective signaling mechanisms of elabela.

The prevention of cardiotoxicity, particularly the primary prevention of cardiomyocyte damage during DOX treatment, remains a key challenge in the field of cardiooncology [40]. The administration of liposomal forms of DOX (Doxil and Myocet) has a beneficial effect on heart minimizing accumulation of the drug in healthy cardiac tissue [41]. However, regardless of the specific form of DOX being used, it remains necessary to implement additional protection strategies. Among the pharmacological strategies, there is the dexrazoxane- the only approved cardioprotective agent [42], drugs that modulate the renin–angiotensin–aldosterone system (enalapril, telmisartan, spironolactone), beta-blockers, statins [43], but also resveratrol [17] and Q10 [18].

Like apelin, elabela acts through the APJ receptor to activate protective cascades (PI3K/AKT and ERK1/2), which reduce OS and prevent apoptosis and cardiomyocyte dysfunction [44]. In our previous study, we investigated electrocardiographic abnormalities induced by DOX in an animal model: ELA40 (40 µg/kg b.w./day ×28) was found to prevent DOX-induced prolongation of the QT and QTc intervals. Additionally, ELA40 was found to shorten the QRS complex duration and increase heart rate. However, we observed the opposite results in the ELA200 group (200 µg/kg b.w./day ×28). It appears that ELA affects cardiac electrocardiographic activity in a dose-dependent manner. Similar observations were made for both doses of apelin (40 and 200) This evidence indicates the strong potential of this peptide to enhance cardiac parameters but in a dose-dependent manner [45].

Elabela, like dapagliflozin (drug from the sodium-glucose transporter type 2 inhibitor group), has been shown to reduce OS in vivo, while dapagliflozin in the DOX cardiotoxicity model additionally reduced lipid peroxidation, improved cholesterol metabolism and stabilized endothelial function [46].

The challenge in determining the potential of elabela in translating results from animal models into clinical practice still remains strong. Factors such as species differences in drug metabolism, cardiac physiology and compensatory mechanisms, limitations of acute and chronic cardiomyopathy models, lack of comorbidities and combination therapy protocols, and insufficient consideration of gender and age all make it difficult to predict treatment efficacy of elabela in patients [47].

Combining elabela with established cardioprotective strategies could improve protection of the cardiomyocytes, but further studies are needed to evaluate the potential side effects and safety of this combination.

Overall, our study adds to the growing body of evidence supporting the role of elabela as a promising cardioprotective agent capable of mitigating the deleterious effects of DOX-induced oxidative injury. Although our analysis focused on classical OS markers, these markers alone do not fully explain the protective effects of elabela that were observed. This suggests that additional pathways may be involved, and this warrants further investigation.

## Figures and Tables

**Table 1:** Scheme of the experiment.

Group	Osmotic Pump (28 days, 2.5 µL/h) Starting from Day 1	Injection (DOX/Saline) Every 7 Days, 4 Doses in Total Starting from Day 1	Animals (n)
1. Control (NaCl)	Saline (0.9% NaCl)	Saline (0.9% NaCl)	8
2. Doxorubicin (DOX)	Saline (0.9% NaCl)	Doxorubicin 3.5 mg/kg body weight. in 1ml of 0.9% NaCl	8
3. Doxorubicin + Elabela (ELA40)	Elabela 40 µg/kg body weight./day in 0.9% NaCl		8
4. Doxorubicin + Elabela (ELA200)	Elabela 200 µg/kg body weight day in 0.9% NaCl		8

**Table 2:** The effect of elabela on the cardiotoxic effects of chronic DOX administration in rats.

Treatment	Degree of Apoptosis
NaCl	−
Doxorubicin	+
Doxorubicin + Elabela 40 µg/kg b.w.	+/−
Doxorubicin + Elabela 200 µg/kg b.w.	−

## Data Availability

The corresponding author, K.K. (Katarzyna Kamińska), will provide the data that supports the findings of this study upon request.

## Supplementary Materials

The following supporting information can be downloaded at: https://www.mdpi.com/article/10.3390/biomedicines13102407/s1. Supplementary Figure S1. Representative calibration plot (standard curve) for MDA, showing absorbance (OD) versus concentration (ng/mL). Supplementary Figure S2. Representative calibration plot (standard curve) for 8-OHdG, showing optical density (OD) versus concentration (ng/mL). Supplementary Figure S3. Representative calibration plot (standard curve) for Elabela, showing optical density (OD) versus concentration (ng/mL). Supplementary Table S1. Heart mass (mg) of rats in four experimental groups. Data are shown for 8 animals per group (n = 8). Values represent mean ± SD (standard deviation). This supplementary table provides individual group data supporting the findings presented in the main text.
