# Supplementary material for: Elabela Attenuates Doxorubicin-Induced Oxidative DNA Damage and Apoptosis in Rat Left Ventricular Myocardium
Source: Biomedicines. 2025 Sep 30;13(10):2407. doi: 10.3390/biomedicines13102407 (PMC12562232; doi:10.3390/biomedicines13102407)
Supplement: Supplementary file 1 [file biomedicines-13-02407-s001.zip › biomedicines-3811007-supplementary.pdf]

Supplementary Materials

The following supplementary figures provide additional data supporting the main findings of the manuscript, specifically include the representative calibration plots (standard curves) for each analyte measured by ELISA showing optical density (OD) versus analyte concentration and mean ± standard deviation allowing comparison of the heart weights of animals.

Supplementary Figure S1. Representative calibration plot (standard curve) for MDA, showing absorbance (OD) versus concentration (ng/ml).

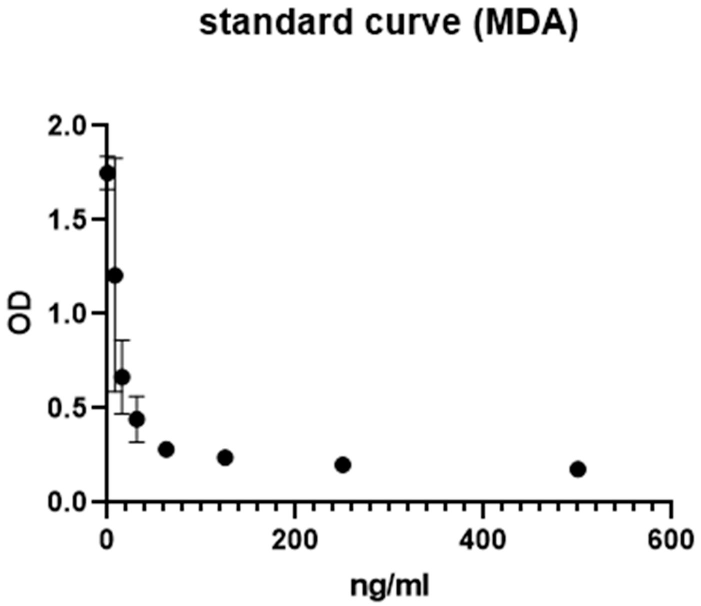

Supplementary Figure S2. Representative calibration plot (standard curve) for 8-OhdG, showing optical density (OD) versus concentration (ng/ml).

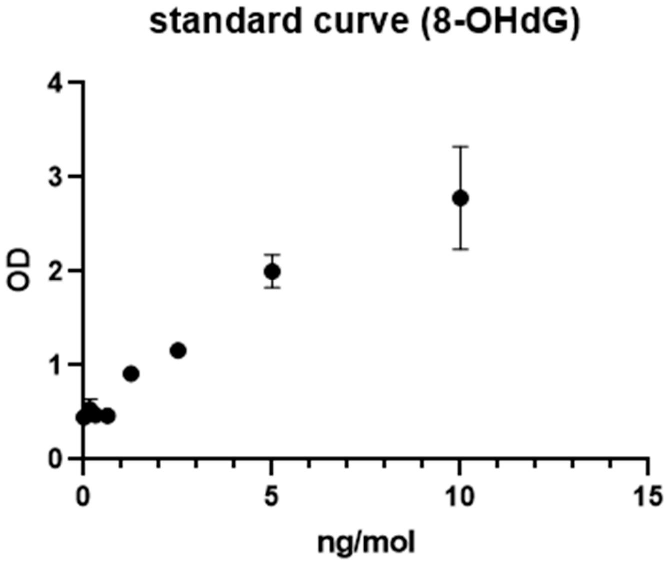

Supplementary Figure S3. Representative calibration plot (standard curve) for Elabela, showing optical density (OD) versus concentration (ng/ml).

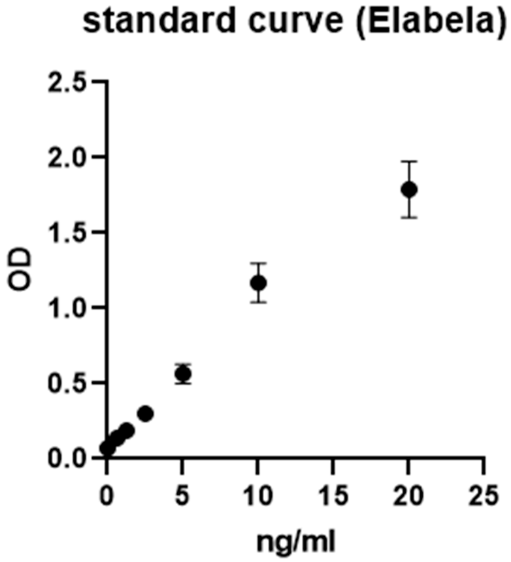

This supplementary table provides individual group data supporting the findings presented in the main text.

Supplementary Table S1. Heart mass (mg) of rats in four experimental groups. Data are shown for 8 animals per group (n = 8). Values represent mean ± SD (standard deviation).

Animal (8 per group)	NaCl	DOX	ELA40	ELA200
1	1589.0	1003.3	1274.5	1131.39
2	1171.6	943.9	849.5	1277.2
3	1909.0	864.2	1157.7	1307.7
4	1325.0	875.6	1072.7	1461.1
5	1340.3	841.8	1026.4	1274.4
6	1309.9	1036.3	1219.9	1305.3
7	1194.2	1193.8	1195.2	1355.0
8	1280.6	1036.6	1412.1	1188.0
**Mean ± SD**	**1390.9 ± 245.0**	**974.4 ± 117.8**	**1151.0 ± 170.2**	**1287.51± 100.0**
